# *In Vivo* Image Analysis of BoHV-4-Based Vector in Mice

**DOI:** 10.1371/journal.pone.0095779

**Published:** 2014-04-21

**Authors:** Valentina Franceschi, Fabio Franco Stellari, Carlo Mangia, Sarah Jacca, Sophia Lavrentiadou, Sandro Cavirani, Mathias Heikenwalder, Gaetano Donofrio

**Affiliations:** 1 Department of Medical-Veterinary Science, University of Parma, Parma, Italy; 2 Institute of Virology, Technische Universität München, Helmholtz Zentrum München, München, Germany; Cedars-Sinai Medical Center; UCLA School of Medicine, United States of America

## Abstract

Due to its biological characteristics bovine herpesvirus 4 (BoHV-4) has been considered as an appropriate gene delivery vector. Its genomic clone, modified as a bacterial artificial chromosome (BAC), is better genetically manipulable and can be used as an efficient gene delivery and vaccine vector. Although a large amount of data have been accumulated *in vitro* on this specific aspect, the same cannot be asserted for the *in vivo* condition. Therefore, here we investigated the fate of a recombinant BoHV-4 strain expressing luciferase (BoHV-4-A-CMVlucΔTK) after intraperitoneal or intravenous inoculation in mice, by generating a novel recombinant BoHV-4 expressing luciferase (BoHV-4-A-CMVlucΔTK) and by following the virus replication through *in vivo* imaging analysis. BoHV-4-A-CMVlucΔTK was first characterized *in vitro* where it was shown, on one hand that its replication properties are identical to those of the parental virus, and on the other that the transduced/infected cells strongly express luciferase. When BoHV-4-A-CMVlucΔTK was inoculated in mice, either intraperitoneally or intravenously, BoHV-4-A-CMVlucΔTK infection/transduction was exclusively localized to the liver, as detected by *in vivo* image analysis, and in particular almost exclusively in the hepatocytes, as determined by immuno-histochemistry. These data, that add a new insight on the biology of BoHV-4 *in vivo*, provide the first indication for the potential use of a BoHV-4-based vector in gene-transfer in the liver.

## Introduction

Bovine herpesvirus 4 (BoHV-4) is a gammaherpesvirus able to replicate in a broad range of host species both *in vivo* and *in vitro* [1]. *In vitro*, BoHV-4 is able to replicate in primary cell cultures and cell lines from various animal species such as cattle, sheep, goat, swine, cat, dog, rabbit, mink, horse, turkey, ferret, chicken, hamster, rat, mouse, and monkey [1]–[7]. In addition to cattle, the BoHV-4 natural host, isolates of BoHV-4 have been recovered from other healthy ruminant species such as zebu (*Bos indicus*), American bison (*Bison bison*), African buffalo (*Syncerus caffer*), sheep and goat. Sporadic isolations were also reported in the lion and the cat. In addition to its natural hosts, BoHV-4 establishes persistent infections also in the rabbit, which therefore, is considered as an experimental host of the virus [8], [9] The BoHV-4-based vector, a recombinant BoHV-4 cloned as bacterial artificial chromosome (BAC) expressing diverse immune-dominant antigens from different pathogens, has been shown to successfully immunize mice [10], rats [11], rabbits [5], sheep [3], swine [6] and goats [12]. Although BoHV-4 has been detected in many tissues, accumulated evidence suggests that the site of persistence both in natural and experimental hosts is represented by the cells of the monocyte/macrophage lineage [8], [9]. To date, the large part of the literature on the biology of BoHV-4 is based on experiments realized *in vitro*, while much less has been obtained from studies *in vivo,* mainly due to the large size of its natural host, which renders any experimentation both difficult and expensive. To overcome to some extent this gap, in the present study we investigated by novel *in vivo* image analysis techniques, transduction and clearances of BoHV-4 in mice experimentally infected with a novel recombinant BoHV-4 (BoHV-4-A-CMVlucΔTK) expressing firefly luciferase.

## Results

### Generation of a Recombinant BoHV-4 Expressing Luciferase (BoHV-4-A-CMVlucΔTK)

**Figure 1:**
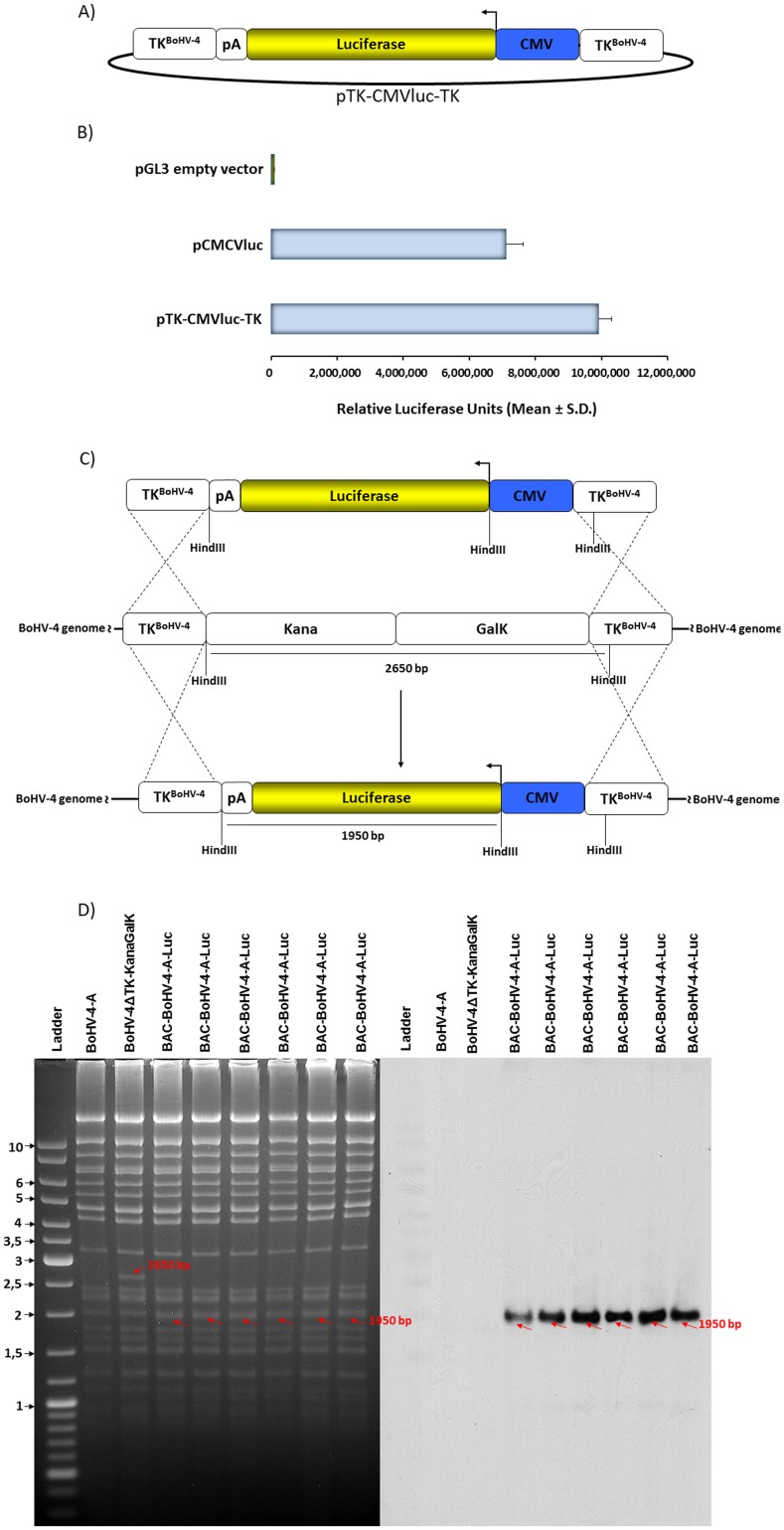
Recombinant virus generation. A) Diagram (not on scale) of pTK-CMVluc-TK construct containing the human cytomegalovirus promoter (CMV; blue), luciferase ORF (Luciferase; yellow), the bovine growth hormone polyadenylation signal (pA; white) and two BoHV-4 thymidine kinase gene flanking sequences (TK; white). B) Transiently transfected HEK cells with pGL3, pCMVluc and pTK-CMVluc-TK vectors at 24 hours. The data represent the mean relative Luciferase units, each reaction was done in quadruplicate and each point represents the mean ± standard error from 3 experiments. Values differ significantly from the pGL3 empty vector control (P≤0.001). C) Schematic diagram (not on scale) of the retargeting strategy employed on BAC-BoHV-4-A genome targeted at the TK locus with a KanaGalK selectable cassette (pBAC-BoHV-4-A-TK-KanaGalK-TK). The Kana/GalK cassette was removed via heat-inducible homologous recombination using the TK-CMVluc-TK targeting fragment and thus replaced with the CMVluc cassette. D) The selected colonies were tested through HindIII restriction enzyme analysis, agar gel electrophoresis and Southern blotting. The retargeted clones were detected based on restriction enzyme mapping, by the disappearance of the 2.650 bp band (indicated by red arrows) and the appearance of a 1.950 bp band, and confirmed by Southern blotting.

**Figure 2:**
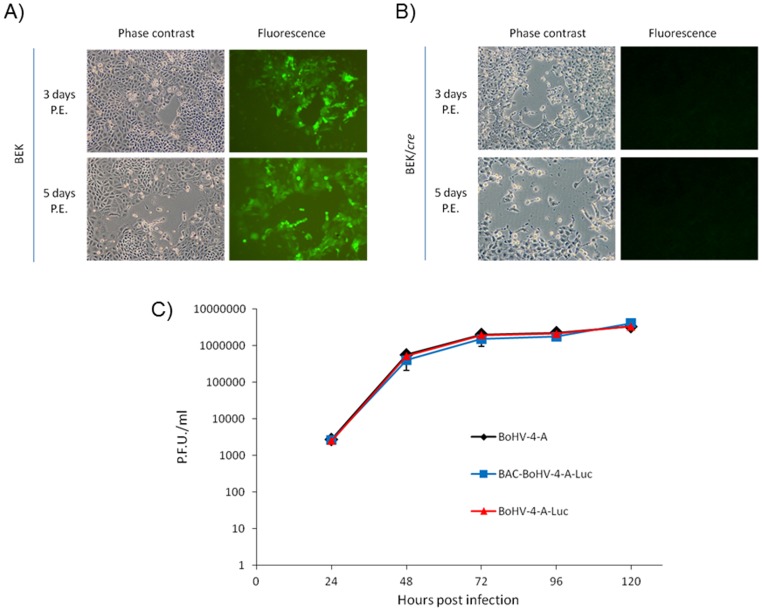
BoHV-4-A-CMVlucΔTK characterization. Representative images [phase contrast and fluorescence; 3 and 5 days post electroporation (P.E.); 10X] of BoHV-4-A-CMVlucΔTK virus reconstitution in BEK (A) and BEK/*cre* (B) cells, following pBAC-BoHV-4-A-CMVlucΔTK electroporation. Although BoHV-4-A-CMVlucΔTK virus could be reconstituted also in BEK cells (A), the floxed BAC plasmid containing a GFP cassette took place only in BEK/*cre*, in fact green plaques were not present. C) Replication kinetics of BoHV-4-A-CMVlucΔTK compared with BAC-BoHV-4-A-CMVlucΔTK (reconstituted virus in BEK cells, were the BAC plasmid was left into the viral genome) and BoHV-4-A. The data presented are the means ± standard errors of triplicate measurements (*P*>0.05 for all time points as measured by Student's *t* test).

The firefly luciferase is the most popular, powerful and efficient reporter enzyme used in bioluminescence [13]. Therefore, the luciferase ORF was chosen as an ideal reporter gene for tracking BoHV-4 tissue tropism and clearance *in vivo*. A luciferase expression cassette, coming from pCMVluc, containing the CMV promoter and the bovine growth hormone polyadenylation signal, was first sub-cloned in the pINT2 shuttle vector [1], a plasmid vector containing two BoHV-4 TK gene sequences, to obtain a luciferase expression cassette flanked by two BoHV-4 TK gene sequences, pTK-CMVluc-TK **(** **Fig. 1A** **)**. Prior to integration of pTK-CMVluc-TK into the TK locus of BoHV-4 genome, its functionality was assessed by transient transfection assay in HEK 293T cells where pTK-CMVluc-TK strongly expressed luciferase as monitored by luminometry **(** **Fig. 1B** **)**. Next the TK-CMVluc-TK cassette was introduced into the TK locus of BoHV-4-A genome **(** **Fig. 1C** **)**. Thus, the TK-CMVluc-TK cassette was excised from pTK-CMVluc-TK and electroporated in SW102 *E. coli* containing the pBAC-BoHV-4-A-KanaGalKΔTK, a Bacterial Artificial Chromosome (BAC) BoHV-4 genome clone whose TK locus has been targeted with a KanaGalK selection cassette [4]. The selected clones were first analyzed by HindIII restriction enzyme digestion and afterwards by southern blotting using a luciferase specific probe. Retargeted clones (BAC-BoHV-4-A-CMVlucΔTK) were well distinguishable from the un-retargeted control clone (BAC-BoHV-4-A-KanaGalKΔTK) either by HindIII digestion, where the 2650 bp band disappeared in the un-retargeted clone and a new band of 1950 bp appeared in the retargeted clone, or by southern blotting **(** **Fig. 1D** **)**. pBAC-BoHV-4-A-CMVlucΔTK stability was checked by serial passages and HindIII digestion assessment **(data not shown)**. Infectious BoHV-4-A-CMVlucΔTK virus was reconstituted in both BEK **(** **Fig. 2A** **)** and BEK*cre* cells expressing the cre Recombinase **(** **Fig. 2B** **)** [4], which enabled the depletion of the floxed BAC cassette from pBAC-BoHV-4-A-CMVlucΔTK. Furthermore, BoHV-4-A-CMVlucΔTK, BAC-BoHV-4-A-CMVlucΔTK and BoHV-4-A were compared in terms of replication capacity, and no differences were observed among them **(** **Fig. 2C** **)**.

### BoHV-4-A-CMVlucΔTK Infected Cells Express Luciferase as Monitored by *in vivo* Image Analysis

**Figure 3:**
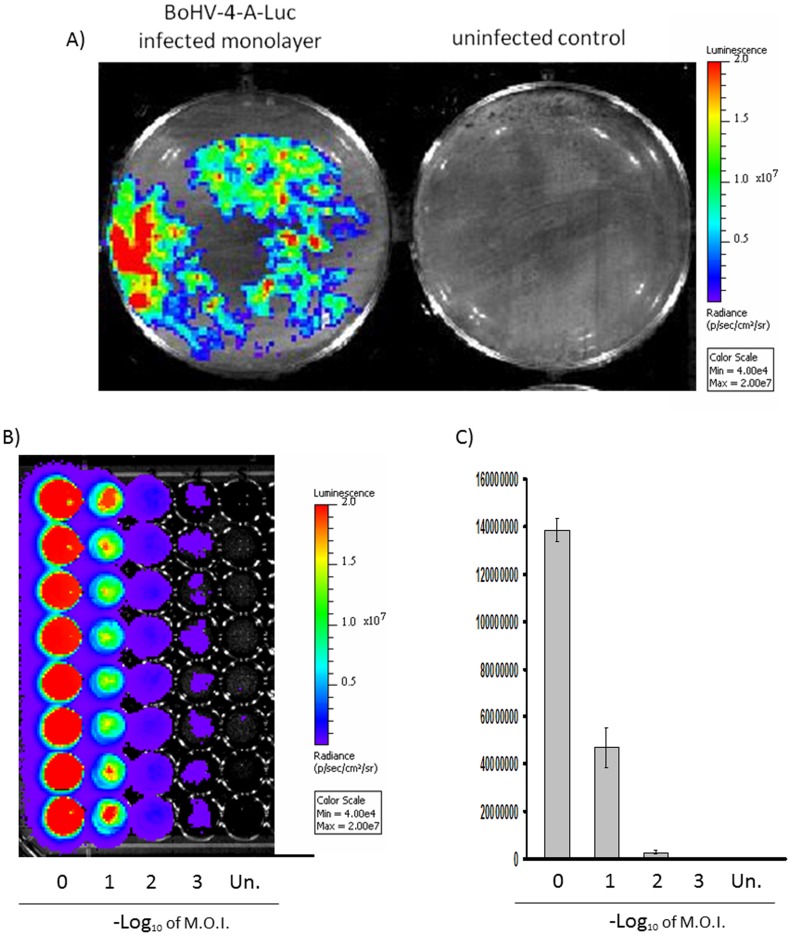
BoHV-4-A-CMVlucΔTK infected cells express luciferase. A) Representative images of BoHV-4-A-CMVlucΔTK infected and uninfected BEK cells monolayers expressing luciferase as observed by BLI. BEK cells infected with different M.O.I. of BoHV-4-A-CMVlucΔTK, visualized by BLI (B) and photons emission quantified by BLI. The data presented are the means ± standard errors of triplicate measurements and each infection was repeated 8 times (*P*>0.05 for all time points as measured by Student's *t* test).

BEK monolayers, containing plaques originating from pBAC-BoHV-4-A-CMVlucΔTK infection, strongly expressed luciferase, as monitored by *in vivo* bioluminescence imaging (BLI) analysis **(** **Fig. 3A** **)**. To better investigate the correlation between BoHV-4-A-CMVlucΔTK infection and the bioluminescence signal, BEK cells were infected with different multiplicity of infection (M.O.I.) of BoHV-4-A-CMVlucΔTK (1, 0.1, 0.01 and 0.001), and bioluminescence was measured 48 h post infection **(** **Fig. 3B and C** **)**. Bioluminescence signal was detectable at doses of infection as low as 0.001 M.O.I. and increased concomitantly with the increase of M.O.I. of BoHV-4-A-CMVlucΔTK.

### *In vivo* BoHV-4-A-CMVlucΔTK Tropism

**Figure 4:**
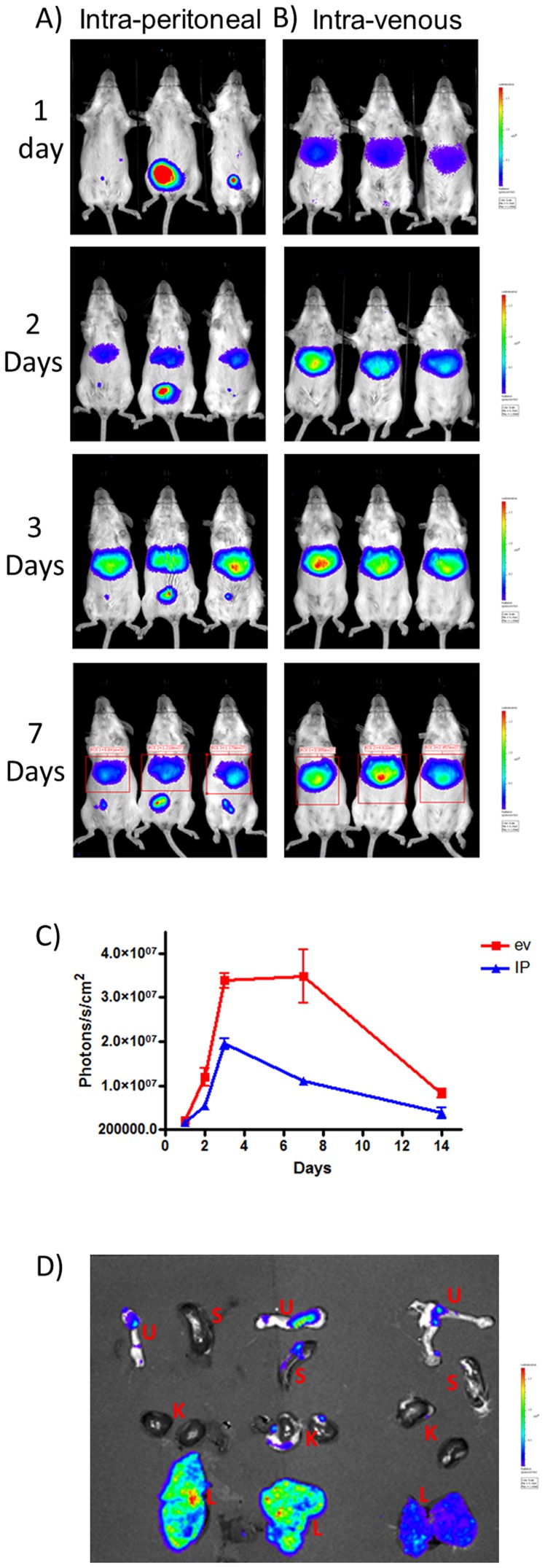
In vivo imaging of BoHV-4-A-CMVlucΔTK inoculate mice. A) Representative images of groups of mice (n = 3 per group) intra-peritoneal (A and blue line in C) or intra-venous (B and red line in C) inoculated with BoHV-4-A-CMVlucΔTK. Mice were monitored at 1, 2, 3, 7 and 14 (not shown) days post inoculation by BLI; light intensity was quantified using the LivingImage software (C). The experiment was repeated three times and each point represents the mean ± standard deviation of 9 animals. Data were expressed as photons/second (S)/cm^2^ and statistical differences were tested by One Way ANOVA followed by Dunnet’s post hoc test for group comparisons. Results are reported as mean ± SD and significance attributed when *P*<0.05 (*) or *P*<0.01 (**). D) Representative BLI image of explanted abdominal organs [uterus (U; in red), spleen (S; in red), kidney (K; in red) and liver (L; in red)] from BoHV-4-A-CMVlucΔTK intra-peritoneal inoculated mice (n = 3) at 3 days post inoculation. The same experiment was repeated 3 times with identical results.

The site of BoHV-4 persistent infection, both in the bovine natural host, and in the rabbit, is represented by the spleen [8], [9]. Moreover, although it has been shown that BoHV-4 does not replicate in mouse or rat brain, some data form the literature indicate that reporter BoHV-4 gene expression has been observed in ependymal cells and the rostral migratory stream area after injection into the lateral ventricle of brains of subjects form these two animal species, without inducing disease [7], [10], [11], [14]–[16]. Taking advantage of these notions and of the availability of BoHV-4-A-CMVlucΔTK, it was of interest to visualize the fate of the virus after systemic injection in mice. Initially 3 mice were intra-peritoneally inoculated with 10^7^ TCID_50_ of BoHV-4-A-CMVlucΔTK and the expression of luciferase was monitored by *in vivo* image analysis at different times post inoculation (1, 2, 3 and 7 days). All the inoculated mice displayed a strong luminescence signal localized in the projection area of the liver **(** **Fig. 4A** **)**. The signal was detectable in not earlier than 2 days post inoculation (∼5×10^5^ Photons/s/cm^2^), peaked at day 3 (∼2×10^7^ Photons/s/cm^2^) and decreased from 7 days post inoculation (∼3×10^5^ Photons/s/cm^2^) **(** **Fig. 4A and D** **)**. When the same dose of virus was intra-venously injected in a second group of 3 mice, although the luminescence signal was again localized in the liver, it was in general stronger in comparison to that monitored in the liver of intra-peritoneally inoculated mice. It was detectable as early as 24 hours post inoculation (∼1×10^7^ Photons/s/cm^2^), and, as in the case of the intra-peritoneally inoculated mice, peaked at day 3 (∼2×10^7^ Photons/s/cm^2^) and decreased from 7 days post inoculation (∼3×10^5^ Photons/s/cm^2^) **(** **Fig. 4A and D** **)**. Following measurements showed that for both intravenous and intraperitoneal BoHV-4-based vector administration, luciferase expression and activity could be well detectable till 60 days post inoculation, which was the end of the observation period **(data not shown)**.

To verify if BoHV-4-A-CMVlucΔTK infects only the liver and no other abdominal organs, a new group of 3 mice were intra-venously inoculated with 10^7^ TCID_50_ of BoHV-4-A-CMVlucΔTK, sacrificed 2 days post inoculation, and their abdominal organs explanted and analyzed by *in vivo* image analysis. As shown in **Fig. 4D**, only the liver was involved by BoHV-4-A-CMVlucΔTK infection/transduction. Further, when other organs like kidney, spleen, lung, heart, blood and lymph nodes were analyzed by quantitative PCR for BoHV-4 DNA presence, no viral DNA could be detected (data not shown).

In order to prove that either intra-peritoneally or intra-venously inoculated BoHV-4-A-CMVlucΔTK homed to the liver, the livers from 3 intra-peritoneally and 3 intra-venously BoHV-4-A-CMVlucΔTK inoculated mice were explanted 7 days post inoculation and titrated on BEK cells for replicating competent BoHV-4-A-CMVlucΔTK. In all livers tested no replicating virus could be revealed **(data not shown)**. As previously reported, no clinical signs were detected in any BoHV-4-A-CMVlucΔTK inoculated animals during the total period of the experimentation. To assess an eventual vector toxicity, two group of 3 mice were intravenously inoculated with either 10^7^ TCID_50_ of BoHV-4-A-CMVlucΔTK or PBS as a control. Collected blood samples, at 7 days post inovulation, were examined for serum transaminase (ALT and AST) levels, which were not significantly different between the subjects of the two groups **(data not shown)**.

### Hepatocytes are Infected/Transduced by BoHV-4

**Figure 5:**
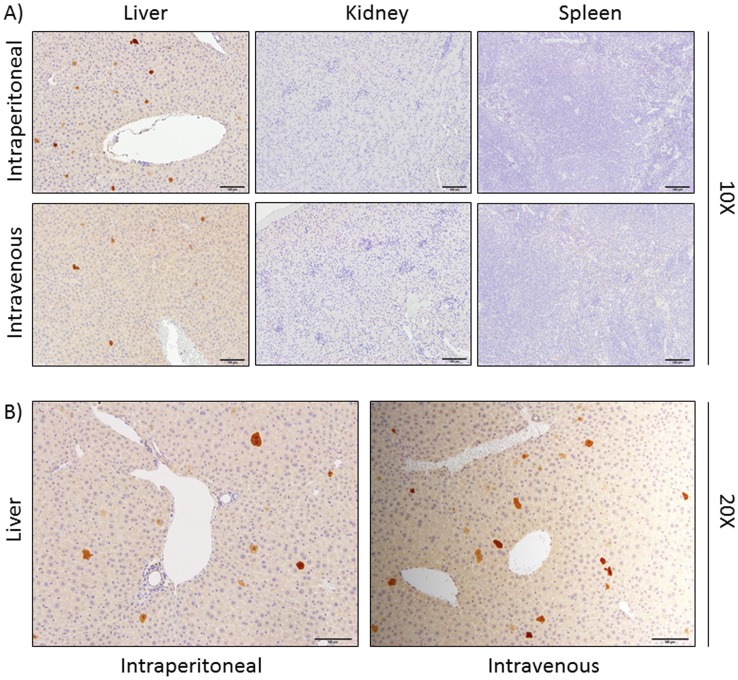
BoHV-4-A-CMVlucΔTK liver cells transduction. Hematoxylin, eosin and immunoperoxidase staining representative images (10X) of liver, kidney and spleen from mice inoculated with BoHV-4-EGFPΔTK at 3 days post inoculation. The dark spots on the liver sections correspond to hepatocytes expressing GFP. The experiment was repeated 3 times and similar results were obtained. B) Higher magnitude (20X) representative images of stained liver from mice inoculated with BoHV-4-EGFPΔTK at 3 days post inoculation.

Although it has been established that viruses can be cleared in the liver by different cell types including the scavengers of the sinusoids, Kupffer cells (KC) and those of the sinusoidal endothelium (liver sinusoidal endothelial cell, LSEC) [17], to date specific cellular elimination pathway adopted for BoHV-4 information is still missing. Therefore in the present study we preliminary investigated the homing of the virus in different liver compartments after inoculation. To this aim, the livers the spleens and the kidneys of a group of 3 mice intra-venously inoculated with BoHV-4-EGFPΔTK, a recombinant BoHV-4 expressing enhanced green fluorescent protein (EGFP) fixed and examined by immuno-histochemistry using a monoclonal antibody against GFP. Surprisingly, GFP was detected only in the hepatocytes of all livers examined but not in the other organs **(** **Fig. 5** **)**.

## Discussion

When a pathogenic virus is experimentally inoculated in a host organism, the infection can be easily detected through several macroscopic parameters such as the death of the host, the appearance of symptoms, the appearance of lesions and/or other clinically measurable specific signs. In the case of moderately or not pathogenic viruses, such as BoHV-4, whose presence and persistence is not directly correlated to a specific pathology, it is extremely difficult to predict and follow the infection outcome, as well as the fate of the virus within the host organism. As previously reported, a recombinant BoHV-4 (BoHV-4EGFPΔTK) expressing EGFP, when inoculated into the lateral ventricle of both wild type [16] and partially immune-compromised mouse brain [10], was able to infect and transduce a cell population of the rostral migratory stream in the complete absence of clinical signs. Therefore, taking into account the peculiar behavior of BoHV-4 directly injected into a specific organ, we investigated here its fate once it is has been systemically inoculated into the blood stream or into the peritoneum. However, before approaching this issue, it was of primary importance to identify a flexible, reliable and sensitive method to track BoHV-4 *in vivo*.

Whole animal bioluminescent imaging (BLI) is progressively becoming more widely applied by investigators with diverse backgrounds because of its low cost, high throughput, and relative ease of operation in visualizing a wide variety of *in vivo* cellular events. In addition, the ability to continually monitor a single individual reduces the amount of inter-animal variation and can reduce error, leading to higher resolution and less data loss. In addition, the constant progress in the hardware and software required for this technique facilitate its application by researchers with little background in molecular imaging on living animals [18]. Having an integrated bioluminescent reporter gene into the genome of a virus not only allows the rapid quantification of viral replication levels but also, upon introduction of the luciferase substrate, gives the noninvasive imaging of infected the tissues. In fact, dynamic whole-body imaging of living animals allows to assess not only where in the body the infection starts but also where it spreads. Therefore, with this in mind, a recombinant BoHV-4 expressing luciferase under the control of the human CMV enhancer promoter, named BoHV-4-A-CMVlucΔTK, was generated. Because an increase of the CMV promoter activity was previously observed in cells infected with BoHV-4, this promoter was chosen to drive the expression of Luciferase [4] in experimentally infected animals. Luciferase expression cassette was inserted into the TK locus because it was previously described that recombinant BoHV-4 with reporter cassettes inserted into the TK open reading frame results in no alteration of viral growth [1], [12]. In fact, BoHV-4-A-CMVlucΔTK growth properties were identical to those of their derivative viruses and cells infected with BoHV-4-A-CMVlucΔTK expressed high levels of Luciferase and the photons emitted from the infected cells were easily measurable as a function of the dose of infection. Probably the most striking observation was made *in vivo*, when BoHV-4-A-CMVlucΔTK was intra-peritoneally or intra-venously inoculated and most of the luminescence signal was localized in the liver. Such a remarkable hepatic tropism of BoHV-4, could be much likely attributed to the vascularization system of the organ and not to the presence of specific ligands, receptors or other attractive molecules. On the other hand, it has been known for more than 50 years that different viruses when injected into the blood stream of an animal are cleared by the liver with astonishing rapidity and efficiency [19]. The liver is the largest and a highly vascularized organ. It is the only organ in the body to have two circularization systems, the systemic with the hepatic artery that brings oxygenated blood directly from the heart and the portal with the portal vein that brings nutrients from the gut and supplies 70% of the blood flow to the liver.

In the present work, 10^7^ BoHV-4-A-CMVlucΔTK particles per injection have been employed. This dose represents a rather low dose respect to the viral vector dose (10^9^–10^12^) used in other works based on adeno or adeno-associated vector and despite of such a high dose a non-linear dose response of liver transduction was obtained [20]. This non-linear dose response resides in the complex cellular composition of the liver. Parenchymal liver cells comprise approximately 67% of resident liver cells whereas sinusoidal cells comprise the remaining cells [21]. Besides endothelial cells, sinusoidal cells comprise Kupffer cells (resident liver macrophages), fat-storing cells (also called stellate cells or Ito cells), and pit cells (natural killer cells). Liver sinusoidal endothelial cells constitute 70%, Kupffer cells 20%, stellate cells 10%, and pit cells less than 1% of the number of sinusoidal cells [22]. Kupffer cells and liver sinusoidal endothelial cells make up the reticulo-endothelial cells of the liver. Kupffer cells account for 80% to 90% of resident macrophages in the entire body [23]. Sinusoidal endothelial cells are scavenger cells that are able to internalize particles up to 0.23 µm under physiologic conditions *in vivo* [24]. Larger particles are taken up by Kupffer cells [24]. Since most gene transfer vectors have a diameter below 0.23 µm, uptake of vectors by both Kupffer cells and liver sinusoidal endothelial cells is a serious obstacle that limits the efficiency of hepatocyte-directed gene transfer [25], [26]. Several investigations have demonstrated that different adenoviral serotypes are rapidly sequestered in the liver after intravenous delivery [27]. Cellular uptake of adenoviral vectors after systemic gene transfer occurs predominantly in non-parenchymal liver cells (*i.e.* mainly liver sinusoidal endothelial cells and Kupffer cells) [28]. It was demonstrated that uptake of vectors by non-parenchymal liver cells (*i.e.* mainly liver sinusoidal endothelial cells and Kupffer cells) inversely correlates with transduction of parenchymal liver cells [28]. Depletion of Kupffer cells and macrophages in the liver by intravenous administration of clodronate liposomes results in significantly increased transgene DNA levels in parenchymal liver cells [28] and in increased transgene expression [28]. Besides clodronate liposomes, pre-administration of polyinosinic acid, a scavenger receptor A ligand, before gene transfer has been shown to prevent sequestration of adenoviral vectors in Kupffer cells and to enhance parenchymal liver cell transduction [29]. The liver transduction we’ve obtained, using only 10^7^ BoHV-4-based vector particles and without intervention to decrease the uptake of the vector in the liver reticulo-endotelial cells, can be considered satisfactory. This mainly for a large size bovine gammaherpesvirus-based vector like BoHV-4.

Histological examination of livers from inoculated mice indicated that BoHV-4-A-EGFPΔTK the infected/transduced liver were hepatocytes and not Kupffer cells (KC) or sinusoidal endothelial cells (liver sinusoidal endothelial cell, LSEC), which are those responsible for the clearance of viral particles [17]. A possible explanation for this surprising behavior could be that the viral fraction sequestered by KC or LSEC lost the transduction ability due to inactivation by the phagocytic activity of those cells, and thus, GFP could not be expressed. Whereas the GFP expressed in the hepatocytes could derive from the remaining viral fraction that escaped KC or LSEC phagocytosis, and thus maintained infection/transduction ability. Although this is an important question that definitely requires an answer, there is no doubt that the BoHV-4-based vector could transduce mouse hepatocytes when it was intra-venously or intra-peritoneally inoculated. This observation, along with the lack of replication of BoHV-4-A-CMVlucΔTK in the liver, paves the way for the potential use of a BoHV-4-A-based vector in liver gene delivery. Liver represents the center of most metabolic pathways, consequently many metabolic diseases originate in this organ and many of these could be treated by genetic approaches. Some of these diseases in humans, such as alpha 1-antitrypsin deficiency, type I tyrosinemia, Progressive Familial Intrahepatic Cholestasis type III or Wilsons’ disease, Crigler-Najjar syndrome type I, ornithine transcarbamylase (OTC) deficiency, type IIa familial hypercholesterolemia and several coagulation defects have a genetic basis, and could be potentially treated by the expression of heterologous genes delivered by viral vectors with a target specificity restricted to the cellular elements of this organ. To date, two approaches are used for liver gene transfer: *in vivo* gene therapy, which is accomplished by delivery of vectors into the blood stream, and *ex vivo* gene therapy, where cells are isolated from a resected liver lobe, genetically modified *in vitro* and then transplanted back into the donor. In terms of viral gene delivery, the liver is an attractive target for *in vivo* gene transfer studies because hepatocytes are readily accessible via the blood stream. However, no clinical treatment of patients with a liver genetic disease has been achieved so far by the intravenous injection of whatever type of vector carrying a specific therapeutic gene; therefore, new liver gene delivery methods are highly on demand. On that basis of the literature, viral vectors are apparently the most efficient tools to achieve gene transfer to the liver *in vivo*and a large panel of viruses has been contemplated for this purpose. Although BoHV-4 is classified as a gammaherpesvirus based on genome sequencing [30], it differs from other gammaherpesvirus members for important biological properties: it exhibits moderate or no pathogenicity and no oncogenicity, it efficiently replicates and causes cytopathic effects (CPE) in a variety of primary cultures and cell lines of various animal species [31] and has a striking tropism toward many human cancer cells [32], [33]. Furthermore, BoHV-4 can accommodate large amounts of foreign genetic material within its genome without any appreciable effect on its replication. For these reasons, it has been proposed as a viral vector for gene delivery and cancer therapy [1]. Keeping all this in mind, the present investigation, that clearly demonstrates the feasibility of using bioluminescence for real-time, repetitive imaging of BoHV-4-based vector infection/transduction in living mice provides the first indication that a BoHV-4-based vector could represent an interesting alternative viral vector for liver gene therapy.

## Materials and Methods

### Cell Lines

Madin Darby Bovine Kidney [(MDBK) ATCC, CCL-22], bovine embryo kidney [(BEK) from Italian Cell Culture collection, Cell Culture Centre Laboratory, Istituto Zooprofilattico Sperimentale, Brescia, Italy; (BS CL-94)] [34], BEK expressing cre recombinase (BEK*cre*) [4] and Human Embryo Kidney 293T [(HEK 293T) ATCC, CRL-11268] cell lines were cultured with complete growth medium [Dulbecco’s modified essential medium (DMEM) (SIGMA) containing 10% fetal bovine serum (FBS, GIBCO), 2 mM of l-glutamine (SIGMA), 100 IU/ml of penicillin (SIGMA), 100 µg/ml of streptomycin (SIGMA) and 2.5 µg/ml of Amphotericin B (SIGMA)] and incubated at 37°C, 5% CO2 in a humidified incubator.

### Viruses

BoHV-4-A-CMVlucΔTK and BoHV-4-EGFPΔTK were propagated by infecting confluent monolayers of BEK, BEKcre or MDBK cells at a multiplicity of infection (M.O.I.) of 0.5 50% tissue culture infectious doses (TCID_50_) per cell and maintained in minimal essential medium (MEM;SIGMA) with 2% FBS for 2 h. The medium was then removed and replaced with fresh MEM containing 10% FBS. When approximately 90% of the cell monolayer exhibited cytopathic effect (CPE) (72 h post infection), the virus was prepared by freezing and thawing cells three times and pelleting the virions through 30% sucrose, as described previously [16]. Virus pellets were resuspended in cold MEM without FBS. TCID_50_ were determined in MDBK cells by limited dilution.

### Plasmids

The 600 bp human cytomegalovirus immediate early promoter (CMV) was amplified by PCR from pEGFP-C1 (Clontech), with a pair of primers (CMV-KpnI-sense, 5′cccggtacctagttattaatagtaatcaat3′; CMV-HindII-anti, 5′cccaagcttggatctgacggttcacta3′) containing a KpnI and a HindIII restriction site, respectively. The amplicon was cut with KpnI/HindIII, sub-cloned in the pGLE3–Basic vector (Promega) in front of the Photinus pyralis luciferase reporter gene and thus the pCMVluc reporter vector was generated. Next, to generate pTK-CMVluc-TK, the CMVluc expression cassette was cut out from pCMVluc with KpnI/BamHI, the ends were blunted with T4 polymerase and the resulting fragment was subcloned in pINT2 [1] cut with SmaI.

### Transient Transfection Assay and Luciferase Assay

Confluent HEK 293Tcells in 6-well plates were transfected with pTK-CMVluc-TK, using the LTX transfection reagent (Invitrogen) as suggested by the manufacturer. The transfection mixture was prepared in DMEM/high glucose (EUROCLONE) without serum and antibiotics and was left on the cells for 6 h at 37°C, 5% CO_2_, in a humidified incubator. After 6 h, the transfection mixture was replaced with complete medium (EMEM, 10% FBS, 50 IU/ml penicillin, 50 µg/ml streptomycin and 2.5 µg/ml Amphotericin B) and the cells were left to recover for 18 h at 37°C, 5% CO_2_ in air, in a humidified incubator. At 24 h post-transfection, cells were lysed and total proteins were extracted to be analyzed by western immunoblotting. Luciferase reporter assay was performed with a Dual Luciferase Reporter Assay System kit (Promega) with minor modifications. In particular, following treatments, cells were washed with PBS, lysed in 100 µl passive lysis buffer by freeze-thawing at −80°C. According to the manufacturer's specifications, 10 µl of the cell lysate was added to 50 µl LAR, and luciferase activity was determined with a Perkin–Elmer Victor^3^ Multilabel Counter (Perkin-Elmer, Milan, Italy). Individual assays were normalized for *Renilla* luciferase activity with a second reading, adding 50 µl of the Stop & Glo substrate.

### Recombineering and Selection

Recombineering was performed as previously described [4] with some modifications. Freshly prepared electro-competent SW102 containing BAC-BoHV-4-A-KanaGalKΔTK [4] were electroporated with the gel-purified TK-CMVluc-TK ∼4.630 kb fragment obtained by cutting pTK-CMVluc-TK, with EcoRI/PvuII (Fermentas). An aliquot of 25 µl of the purified fragment was used for each electroporation in a 0.1 cm cuvette at 25 µF, 2.5 kV and 201 Ω. After electroporation, the bacteria were recovered in 1 ml LB Broth (BD Biosciences) (15 ml Falcon tube) for 1 h in a 32°C shaking water bath. For the counter selection step, the bacteria were transferred in 10 ml LB in a 50 ml baffled conical flask and incubated for 4.5 h in a 32°C shaking water bath. Serial dilutions of the bacteria were plated on M63 minimal medium plates containing 15 g/l agar (Invitrogen), 0.2% glycerol (SIGMA), 1 mg/l d-biotin(SIGMA), 45 mg/l l-leucine (SIGMA), 0.2% 2-deoxy-galactose (DOG, SIGMA) and 12.5 µg/ml chloramphenicol (SIGMA). Plates were incubated 3–5 days at 32°C. Several selected colonies were picked up, streaked on McConkey agar indicator plates (DIFCO, BD Biosciences) containing 12.5 µg/ml of chloramphenicol and incubated at 32°C for 3 days until white colonies appeared. White colonies were grown in duplicate for 5–8 h in 1 ml of LB containing 50 µg/ml of kanamycin (SIGMA) or LB containing 12.5 µg/ml of chloramphenicol. Only those colonies growing on chloramphenicol and not on kanamycin were kept and grown overnight in 5 ml of LB containing 12.5 µg/ml of chloramphenicol. BAC DNA was purified and analyzed through HindIII restriction enzyme digestion for pTK-CMVluc-TK fragment targeted integration. Original detailed protocols for recombineering can also be found at the recombineering website (http://recombineering.ncifcrf.gov).

### Viral Growth Curves and Plaque Assay

BEK cells were infected with BoHV-4-A, BAC-BoHV-4-A-CMVlucΔTK and BoHV-4-A-CMVlucΔTK at a MOI of 1 TCID_50_/cell and incubated at 37°C for 4 h. Infected cells were washed with serum-free EMEM and then overlaid with EMEM containing 10% FBS, 2 mM l-glutamine, 100 IU/ml penicillin, 100 µg/ml streptomycin and 2.5 µg/ml Amphotericin B. The supernatants of infected cultures were harvested after 24, 48, 72, 96 and 120 h, and the amount of infectious virus was determined by limiting dilution on BEK cells.

### Experimental Animals

Female inbred FVB (7–8 week-old) mice were purchased from Harlan Laboratories Italy (San Pietro al Natisone, Udine). Animals were maintained under conventional housing conditions. Prior to use, animals were acclimatized for at least 5 days to the local vivarium conditions (room temperature: 20–24°C; relative humidity: 40–70%), having free access to standard mouse chow and tap water. All experiments were carried out in rodents and exclusively included painless suppression of animals. The experiments comply with the Principles of Animal Care (publication no. 85–23, revised 1985) of the National Institutes of Health and with the current law of the European Union and Italy (D. L.vo 116/92). The present project was approved by the Ethical Committee of the University of Parma (Italy).

### *In vivo* Bioluminescence Imaging (BLI)

*In vivo* imaging was performed using an IVIS imaging system (Caliper Life Sciences, Alameda, CA). Inoculated mice were imaged using bioluminescence (BLI) after 1, 2, 3, 7 and 14 days following intraperitoneal injection of 150 mg/kg luciferin. The mice were anesthetized with gas anaesthesia (isoflurane 2.5%), imaged for 5 minutes, 10 minutes and 15 minutes after luciferin injection in order to minimized the pharmacokinetic variability among mice. Photons emitted from specific regions were quantified using Living Image software (Caliper Life Sciences, Alameda, CA).
